# Supplementary material for: A membrane insertion code for intrinsically disordered proteins
Source: eLife. 2026 Aug 26;15:RP111515. doi: 10.7554/eLife.111515 (PMC13516693; doi:10.7554/eLife.111515)
Supplement: Peer review history

# Editor report: eLife Assessment

**DOI:** 10.7554/eLife.111515.3.sa0

**Authors:** Heedeok Hong

**Affiliations:** Michigan State University United States

**Keywords:** Convincing, Important

This study provides an **important** advance in understanding how disordered proteins interact with cell membranes by identifying the sequence rules that enable aromatic residues to penetrate deeply into the membrane interior. The integration of complementary computational approaches, including molecular simulations, large-scale sequence analysis, and the development of an online prediction server, makes the work potentially impactful for the membrane protein and intrinsically disordered protein communities. The evidence supporting the main conclusions is generally **convincing**.

# Referee report: Reviewer #1 (Public review)

**DOI:** 10.7554/eLife.111515.3.sa1

Summary:

This work investigates the membrane insertion of aromatic-centered sequences in IDPs. Using a combination of all-atom MD simulations, the PPM method, and development of the sequence-based predictor AroMIP, the authors aim to establish a quantitative membrane insertion role for aromatic-centered motifs. The study demonstrates that flanking aliphatic and basic residues promote membrane insertion, whereas acidic and polar residues suppress insertion, and further reveals a difference between F/W-centered motifs and Y-centered motifs. The resulting AroMIP model achieves high predictive accuracy on human IDPs and is implemented as a publicly accessible web server.

Strengths:

This work addresses an important biological problem, as aromatic-driven membrane insertion remains poorly characterized despite mediating diverse functions like membrane remodeling and signaling. A key strength is the combination of complementary approaches, e.g., MD simulations provide mechanistic insight into insertion pathways, while PPM enables exhaustive sequence space exploration. The large-scale analysis clearly establishes L and R as promoters and E, N, and G as suppressors. The work also provides valuable mechanistic insight into how aromatic, aliphatic, and basic residues cooperate to stabilize membrane insertion states. Another important strength is the development of AroMIP as a practical prediction tool with a user-friendly online server that appears computationally efficient and broadly accessible to the community. The work is also well connected to prior experimental and computational literature, and the authors carefully position their findings within existing knowledge of membrane-associated IDPs.

Comments on revised version:

I think the authors have addressed all my concerns. I do not have further comments or requests for additional revisions. Thank you for all the hard work!

# Author comment: Author response

**DOI:** 10.7554/eLife.111515.3.sa2

**Authors:** Fidha Nazreen Kunnath Muhammedkutty, Huan-Xiang Zhou

**Affiliations:** University of Illinois Chicago Chicago United States; University of Illinois Chicago Chicago United States

The following is the authors’ response to the original reviews.

**eLife Assessment**

This study provides a valuable advance in understanding how disordered proteins interact with cell membranes by identifying the sequence rules that enable aromatic residues to penetrate deeply into the membrane interior. The integration of complementary computational approaches, including molecular simulations, large-scale sequence analysis, and the development of an online prediction server, makes the work potentially impactful for the membrane protein and intrinsically disordered protein communities. The evidence supporting the main conclusions is generally convincing, although its transferability across diverse membrane compositions and its validity as a prediction tool for real protein-membrane systems remain to be further established.

We thank the editors for recognizing our study as a valuable advance. This work lays a solid foundation for future developments to account for diverse membrane compositions and further refinements after additional experimental tests.

**Public review:**

**Reviewer #1 (Public review):**

A primary limitation is the heavy reliance on computational modeling. Training for AroMIP is generated using PPM rather than direct experimental measurements, and so the model may primarily reproduce PPM behavior rather than true membrane insertion thermodynamics. Moreover, all simulations use a single lipid composition (POPC:POPS:_2_ 70:25:5), but biological membranes vary substantially in cholesterol, cardiolipin, and acidic lipid content. Whether AroMIP's predictions transfer to diverse lipid environments remains untested. The 5% _2_ concentration used in the simulations is higher than that of a normal mammalian cell and may therefore overemphasize electrostatic contributions. Applicability beyond short 9-residue motifs is unclear, as longer-range interactions or secondary structure in full-length IDRs could modulate insertion in ways the current model does not capture. This could be considered for future development.

The reviewer’s point on our reliance on PPM for training, a single lipid composition, and potential effects beyond a 9-residue motif is well taken. Regarding PPM, we chose it as the optimal compromise for high-throughput data. However, we complemented the high-throughput PPM data with experimental data on an initial set of 10 peptides. Moreover, we validate AroMIP on an additional 12 IDRs (intrinsically disordered regions; Table S2). On membrane composition, we now acknowledge the limitation of our work based on a single composition and point to future developments of AroMIP involving membrane-specific parameterization (p. 19, 3rd paragraph).

On potential effects beyond a 9-residue motif, we now add justification and note neglected factors for future developments (paragraph running from p. 19-20), as suggested by the reviewer.

**Reviewer #2 (Public review):**

(1) Aromatic residues have been shown to partition preferentially to the headgroup region of the lipid bilayer. Most of the papers on this problem were published in the mid 1990s to early 2000s. Some of the most important papers in this regard are the following: von Heijne, Annu. Rev.

Biophys. Biomol. Struct. 1994, 23, 167-192; Doyle et al. Science 1998, 280, 69-77; Landolt-Marticorena, et al. J. Mol. Biol. 1993, 229, 602-608; Killian & von Heijne, TIBS 2000, 25, 429434; Marx & Fleming J. Am. Chem. Soc. 2021, 143, 764-772. Strangely enough, none of these articles is cited.

We have now citations to the Landolt-Marticorena paper and the von Heijne reviews [refs 25-27]. The Doyle paper is not particularly relevant. As for the Fleming paper, we cited a 2016 JACS paper (original ref 27; now ref 30) that specifically dealt with aromatic residues.

(2) This is the most important point and the most serious weakness. The authors find that the PPM method is able to reproduce the results from MD simulations, and the AroMIP model is able to perform well in comparison with PPM and MD, after training AroMIP on a large set of IDR sequences (intrinsically disordered protein regions) of the human proteome. The defining feature of the AroMIP calculation is the recognition of the importance of flanking residues in the membrane-insertion propensity of a sequence containing a central aromatic residue. All this sounds good. However, this is all theoretical. There is no connection to experiment or to any method that draws from experiment. The entire approach relies on the assumption that the MD simulations produce the correct results. There is no proof of the correctness of anything. As one of the greatest physicists of our times, Richard Feynman, wrote, "The test of all knowledge is experiment. Experiment is the sole judge of scientific "truth"."

We emphasize that we have presented substantial experimental support for AroMIP. It correctly predicts the membrane insertion status of the initial set of 10 peptides, which were characterized experimentally. In addition, we validated AroMIP on an additional set of 12 IDRs (Table S2), most of which were characterized by experimental techniques including solution and solid-state NMR, fluorescence, H/D exchange, and cryo-EM. Lastly, we now show good correlation between our insertion scores and binding free energies calculated from the scale determined experimentally by White and co-workers (new Figure S10; p. 15, second paragraph).

(3) The drawings in Figures 2 and 3 are incorrect and misleading. The size of the Tryptophan side chain is about 5.5 Å, whereas one-half of the bilayer ("a monolayer") thickness is about 15 Å. But in the figures, the lipid length and the Trp side chain seem about the same size. This is incorrect even in a qualitative sense.

We have now revised these figures.

**Reviewer #3 (Public review):**

(1) Membrane composition and lipid shape characteristics: The authors chose to use a model membrane bilayer of a distinct lipid composition, POPC: POPS: PI4,5P2 (70:25:5 molar ratio), for their all-atom simulations of the various model peptides. While this may be pertinent for some of these peptides, it is not for many, such as sequence 2 derived from Drp1, which preferentially binds target conical lipids such as cardiolipin (CL) and phosphatidic acid (PA). The rationale behind using PI4,5P2, which can induce positive membrane curvature when sequestered, versus CL and PA, which both induce negative membrane curvature, is not explained.

We now acknowledge the limitation of our work based on a single composition and point to future developments of AroMIP involving membrane-specific parameterization (p. 19, 3rd paragraph). In this Discussion paragraph, we also speculate that conical lipids, by promoting membrane defects, may facilitate membrane insertion.

(2) Parallel vs. perpendicular peptide orientation of sequence 2 in peripheral Drp1-lipid interactions: On page 11, the authors state that their simulation results of sequence 2 derived from Drp1 "contrasts with a transmembrane orientation proposed by Mahajan et al." However, upon review, a transmembrane orientation for this region has never been proposed anywhere. Drp1 is a peripheral membrane protein that reversibly binds CL- and PA-containing membranes via its intrinsically disordered variable domain containing an aromatic-centered WRG motif. Indeed, the model presented in Figure 9 of Mahajan et al. displays a peripheral and parallel orientation of the transiently helical WRG-containing motif rather than a transmembrane (i.e., across the bilayer) orientation. While the authors can distinguish between a parallel vs. perpendicular orientation of this sequence relative to the plane of the membrane bilayer surface from their simulations, suggesting that previous studies indicated a transmembrane orientation for Drp1 is disingenuous and misleading. The term "transmembrane" should be removed or replaced, as it presents a wrong image.

We have now deleted the sentence mentioning “transmembrane orientation”.

(2) Mutational analysis of W vs. F in membrane insertion of W-centered insertion motifs and vice versa: The PPM-based workflow suggests that F-centered sequences have the highest membrane insertion properties as opposed to W-centered ones. A W552F mutation in the WRGML sequence of Drp1 was, however, found to impair function. How do the authors rationalize this? A crossmutational analysis of W vs. F in W-centered motifs and F-centered motifs is warranted.

AroMIP predicts a membrane insertion propensity of 0.782 for the WRGML sequence and a moderately higher propensity, 0.837, with a W552F mutation. This increase contradicts the experimental observation of a 3.6-fold increase in membrane binding affinity by Mahajan et al. We now speculate that the specific lipid, cardiolipin, as the reason for the discrepancy (p. 19, 3rd paragraph). This discrepancy provides a concrete example for the need to account for membrane composition in future developments.

**Recommendations for the authors:**

**Reviewing Editor Comments:**

(1) The membrane composition used in this study is highly specific. The manuscript would benefit from a clearer justification of the lipid composition and discussing the transferability of the approaches to other relevant membrane systems.

We now acknowledge the limitation of our work to a single composition (p. 19, 3rd paragraph). This composition was chosen because it is widely used in both computational and experimental studies (e.g., PMID: 21144818; 21344950; 29845130; 29995324; 34813727; 37406927). However, as we now point out, future developments should account for the effects of lipid composition.

(2) This work heavily relies on computational modeling. Thus, it remains unclear to what extent the model captures the thermodynamics of membrane insertion, rather than reproducing the behavior of the PPM framework. Further comparisons with available experimental results will make this work more impactful.

We note that the manuscript already has substantial experimental support. It correctly predicts the membrane insertion status of the initial set of 10 peptides, which were characterized experimentally. In addition, we validated AroMIP on an additional set of 12 IDRs (Table S2), most of which were characterized by experimental techniques including solution and solid-state NMR, fluorescence, H/D exchange, and cryo-EM. Lastly, we now show good correlation between our insertion scores and binding free energies calculated from the scale determined experimentally by White and co-workers (new Figure S10; p. 15, second paragraph).

(3) Overall, the manuscript is lengthy. Shortening will improve the readability, clarity, and accessibility to a broad audience.

We have shortened some text, as explained below.

**Reviewer #1 (Recommendations for the authors):**

(1) The manuscript uses a single membrane composition… The high PIP_2_ content (5%) in the simulations may overemphasize electrostatic contributions from basic residues. Please discuss how different membrane compositions (e.g., lower _2_, presence of cholesterol, cardiolipin in mitochondrial membranes) might alter the q parameters and whether AroMIP predictions would change qualitatively or quantitatively.

We now discuss how different membrane compositions may alter q parameters (p. 19, 3rd paragraph). We believe that these alterations will change our predictions quantitatively but not qualitatively, given that our validation is against experimental results acquired on a variety of membrane compositions.

(2) The limitations of the current framework should be discussed more explicitly. For example, the applicability of AroMIP beyond isolated 9-residue motifs remains unclear. In full-length IDPs, membrane insertion may be modulated by longer-range sequence interactions, transient secondary structure formation, multivalent interactions, or post-translational modifications.

We now discuss the limitation of the 9-residue motif, and note these neglected factors for future developments (paragraph running from p. 19-20).

(3) The AroMIP web server is user-friendly… The utility of the server could be enhanced by enabling visualization of full-length disordered proteins. For example, if users input a >300 residue IDP, the server could output residue-wise or sliding-window membrane insertion propensity profiles.

We have revised the web server. We now display insertion propensity profiles as a plot and have added a link for users to download.

(4) The manuscript is quite long and in several sections overly descriptive, e.g., in the first MD Results section and portions of the "Additional test cases" section.

We have shortened the first subsection in Results and placed the expanded presentation in Supporting Information. However, we have kept the “Additional test cases” subsection, because Reviewer 2 appears to have overlooked the experimental validation of our method in these additional test cases.

(5) The authors used the Berendsen barostat… known not to reproduce correct volume fluctuations and is generally considered less rigorous for equilibrium simulations, although this does not affect the main conclusions of the work. For future studies, the authors may consider using more modern barostats.

Thank you for the suggestion! We will definitely be using the more modern barostats in future studies.

**Reviewer #2 (Recommendations for the authors):**

(1) The idea of the article seems very interesting. The problem of membrane association mediated by aromatic residues is definitely worth studying. Aromatic residues, especially Tryptophan (W), but also, albeit to a lesser extent, Phenylalanine (F), and Tyrosine (Y) are well known to partition preferentially to the headgroup region of the lipid bilayer. Some of the most important papers in this regard are the following: von Heijne, Annu. Rev. Biophys. Biomol. Struct. 1994, 23, 167-192; Doyle et al. Science 1998, 280, 69-77; Landolt-Marticorena, et al. J. Mol. Biol. 1993, 229, 602608; Killian & von Heijne, TIBS 2000, 25, 429-434; Marx & Fleming J. Am. Chem. Soc. 2021, 143, 764-772. Strangely enough, however, none of these articles is cited. Have the authors read them?

We now cite the relevant references as explained above.

(2) The authors propose to decipher the sequence code for insertion of sequences containing aromatic residues in the membrane employing three types of calculation methods with decreasing order of detail and complexity, but increasing order of efficiency. First, all-atom MD simulations; second, the PPM method (protein positioning in membranes) from Lomize et al (2006), Protein Sci 15, 1318; and third, AroMIP, a mathematical model developed by the authors. Incidentally, I don't see anywhere in the text what AroMIP stands for. Is it Aromatic Membrane Insertion Prediction, or something like that? Please define. In any case, the proposed endeavor is commendable.

We now spell out the acronym at its first occurrence in the Abstract and Introduction (Aromatic Membrane Insertion Predictor).

(3) This is the most important point and the most serious weakness. The authors find that the PPM method is able to reproduce the results from MD simulations, and the AroMIP model is able to perform well in comparison with PPM and MD, after training AroMIP on a large set of IDR sequences (intrinsically disordered protein regions) of the human proteome. The defining feature of the AroMIP calculation is the recognition of the importance of flanking residues in the membrane-insertion propensity of a sequence containing a central aromatic residue. All this sounds good. However, this is all theoretical. There is no connection to experiment or to any method that draws from experiment. The entire approach relies on the assumption that the MD simulations produce the correct results. There is no proof of the correctness of anything. As one of the greatest physicists of our times, Richard Feynman, wrote, "The test of all knowledge is experiment. Experiment is the sole judge of scientific "truth"." Thus, there must be a comparison with experiment, as elaborated in the next point.

We emphasize that we have presented substantial experimental support for AroMIP. It correctly predicts the membrane insertion status of the initial set of 10 peptides, which were characterized experimentally. In addition, we validated AroMIP on an additional set of 12 IDRs (Table S2), most of which were characterized by experimental techniques including solution and solid-state NMR, fluorescence, H/D exchange, and cryo-EM.

(4) I understand that the authors are computational or theoretical physical chemists and would not expect them to perform the experiments. However, there are plenty of data in the literature that can be used to corroborate the calculations. First and foremost, though, the authors must calculate the binding constants for their set of peptides and then compare them with experiment, or use a different set of peptides for which the experimental results are available, and test how PPM and AroMIP perform on those peptides. There are two possible approaches to calculate the binding constants. First, calculate the Gibbs energy of binding from simulation or calculation, and, from that, calculate the binding constant via the Boltzmann factor. Second, and better, is to calculate the probability of binding in the simulations or calculations from the fraction of time that the peptide spends bound to the membrane or in water. According to the ergodic principle, the ratio of the two times is the binding constant. I understand that most amphipathic peptide sequences whose binding constants to membranes have been determined by experiment are long. But some are not. For example, Mastoparan X is a 14-residue antimicrobial peptide, and its dissociation constant from POPC vesicles is known to be about 300 μM.

We would like to make it clear that the aim of our study is to predict membrane insertion propensities of aromatic-centred motifs, not the membrane binding affinity of peptides. These two properties are related but require different ways of validating predictions. For membrane insertion, validation requires that not only the motifs are bound to membranes but also the aromatic side chains are placed in the acyl chain region. Our validation of the 12 IDRs listed in Table S2 targeted these requirements.

That said, we note that free energy of binding and probability of binding calculations, mentioned by the reviewer, have been reported previously, including the free-energy cost of Ala substitutions of aromatic residues located at various depths reported by Waheed et al. (ref 35) and residue-specific insertion depths reported by Wang et al. (ref 13). Both of these studies highlighted the propensities of aromatic side chains in inserting into the acyl chain region.

(5) Furthermore, the entire Wimley-White interfacial hydrophobicity scale was determined using pentapeptides, measuring the equilibrium binding constant to POPC membranes experimentally and then using those data to build a residue-based Gibbs energy of binding (Wimley & White, Experimentally determined hydrophobicity scale for proteins at membrane interfaces. Nature Struct. Biol. 1996, 3, 842-848; White & Wimley Membrane protein folding and stability: Physical principles. Annu. Rev. Biophys. Biomol. Struct. 1999, 28, 319-365.) This allows for the calculation of the binding affinity for any sequence. The calculations have been compared to experiment and shown to have a high accuracy. A note of caution: Be careful, though, because Wimley and White used a mole fraction concentration scale, which makes the Gibbs energy of binding more favorable than the value calculated using the more common molar concentration scale by -2.4 kcal/mol. Calculating the binding constant for the peptides used by the authors from the WW interfacial scale and comparing the results with the authors' results would be a good place to start.

This is an excellent suggestion! We now compare our insertion scores with the binding free energies calculated from the WW interfacial scale (new Figure S10; p. 15, 2nd paragraph). Interestingly, we found moderately higher correlations with binding free energies calculated from the octanol scale, which we suggest is more in line with our insertion scores since octanol mimics the hydrophobic region as suggested by White and Wimley in their 1999 Annu Rev paper.

(6) When we speak of insertion in a bilayer, we normally mean insertion in the nonpolar core, not in the interfacial region, which is what the authors mean in this paper. This is extremely misleading and should be changed, namely in the title, but also throughout the paper.

Actually, by insertion we precisely mean into the nonpolar core, NOT the interfacial region. Throughout the Introduction, when we used the word “insert”, we added “into the acyl chain region” (p. 3, line 6 from bottom; p. 5, lines 6-7 from top and line 7 from bottom; p. 6, line 4 from top). To avoid any confusion, we now also explicitly add “into the membrane hydrophobic core” when the word “insertion” first occurs in the Abstract and in the opening paragraph of Introduction (replacing the previous “deep insertion”).

(7) What is q? It appears in equation (1) on page 14, and is referred to several times afterwards, but it is never defined.

We now elaborate, in the text above and below equation (1), on the meaning of the q parameters: they represent the contributions of flanking residues to the insertion score of a central aromatic residue.

(8) The drawings in Figures 2 and 3 are incorrect and misleading. The size of the Tryptophan side chain is about 5.5 Å, whereas one-half of the bilayer ("a monolayer") thickness is about 15 Å. But in the figures, the lipid length and the Trp side chain seem about the same size. This is wrong even in a qualitative sense.

We have revised these figures.

(9) In Figure 1, the location of the bilayer midplane should be indicated, for example, with a line. Currently, there is a red line on the figures, but that is not the bilayer midplane. A reader may easily - and is likely to - misinterpret the figure.

As explained in the Figure 1B, C caption, the red line is drawn at Z = -3.1 Å, which is the mean position of glycerol C2 carbon atoms (setting Z = 0 for the phosphate plane) and thus the start of the acyl chain region.

**Reviewer #3 (Recommendations for the authors):**

(1) Please refrain from using the term "transmembrane" when describing Drp1-membrane insertion, as Drp1 is a soluble, peripheral membrane-binding protein that reversibly associates with membranes.

We have removed the sentence mentioning “transmembrane orientation”.
